# Properties and Dosage of Chloranodyne

**Published:** 1886-11-20

**Authors:** 


### PROPERTIES AND DOSAGE OF CHLORANODYNE.

Nothing demands the exercise of more skill and intelligence on the part of physicians than the regulation of the dose of a medicine to suit the individual case, the manifestations of the disease, and the idiosyncracies of the patient.

Many valuable medicines often fail to produce their natural therapeutic effect through failure to recognize the importance of the fact that, a proper regard being had to the nature of a drug, its therapeutic and toxic action, it should be given to produce a certain effect, the dose naturally varying with the susceptibility of the patient.

This is especially true of anodynes, a class of medicines than which no other are oftener indicated, and which often fail of their natural effect when not intelligently given.

While physicians are familiar with the range of action and application of officinal anodynes, there are certain formulæ which have been largely employed empirically and with the most gratifying success that many hesitate to employ with the same "prudent boldness," if we may use the term, as they would use the remedies composing it, uncombined.

Such a formula is chloranodyne, which is a scientific substitute for and improvement upon the well-known proprietary preparation, Chlorodyne. The formula of chloranodyne is as follows :

Each gramme (ordinary adult dose) contains—

| | |
|---|---|
| Morphia muriate | .0060 gramme, |
| Tinct. cannab. ind | .0800 gramme, |
| Chloroform | .1350 gramme, |
| Oil of peppermint | .0025 gramme, |
| Tinct. capsicum | .0025 gramme, |
| Hydrocyanic acid, dilute | .0170 gramme, |
| Alcohol | .3000 gramme, |
| Glycerine | .4570 gramme, |

Its effects are characterized by four phases—

1st. A few moments after administration, a gentle heat is experienced at the stomach, succeeded by relief from pain, followed by diaphoresis.

2d. A tranquil, composed state gradually takes place, with or without sleep.

3d. A comfortable sleep, without coma, comes on, allowing the patient to be aroused at any moment, and continuing for some time, during which a favorable change takes place.

4th. The pulse increases, from a small, weak, thready, hurried or bounding one, to a full, yielding, elastic, natural one, decreasing in frequency of beats as well as resistance.

Nausea may occur from two causes : 1st. Excessive dose ; 2d. From constipation. The first may be relieved by 20 drops of aromatic spirits of ammonia ; the second by an aperient.

As a remedial agent in the treatment of febrile, inflammatory or neuralgic affections, its administration has been found to exercise most remarkable curative effects in all stages of disease. The most intense inflammation, equally with neuralgic pains, alike become subdued. It has been found to succeed even in a few doses, when quinine in ½-drachm doses, opium, morphine, and chloroform produced no curative result whatever—a fact which must establish its repute as one of extreme utility.

In regulating the dose, regard must be had to the age of the patient, nature of the disease, and effect required to be produced. It must be remembered that no particular or positive rule can be laid down whereby a certain result must follow from a specified quantity; the scale given is, at most, only a comparative one for safe purposes. When the action of the remedy does not appear to succeed from the ordinary dose it must be increased.

SCALE OF ADULT DOSES.

As a diaphoretic, 5 to 10 drops (to produce perspiration), in coughs, colds, influenza, etc.

As an anodyne, 5 to 15 drops (to relieve pain) in head and heart affections and all neuralgic and rheumatic pains.

As an anti-spasmodic, 10 to 12 drops (to control spasms), for hysteria, rheumatism, gout, delirium tremens and cramps, hæmoptysis, croup, cystic, renal and uterine diseases.

As an astringent, 15 to 30 drops, for cholera, dysentery, diarrhœa, colic, etc.

SCALE OF INFANTILE DOSES.

| Age. | Dose. |
|---|---|
| 1 month to 3 months | 1 to 2 drops. |
| 3 months to 6 months | 2 to 4 drops. |
| 5 months to 12 months | 4 to 5 drops. |
| 1 year to 3 years | 3 to 6 drops. |
| 3 years to 5 years | 3 to 8 drops. |
| 5 years to 8 years | 4 to 10 drops. |
| 8 years to 12 years | 8 to 12 drops. |
| 13 years to 16 years | 10 to 20 drops. |

